# Development of an Innovative Telerehabilitation System to Improve Handgrip Strength

**DOI:** 10.5195/ijt.2022.6497

**Published:** 2022-12-13

**Authors:** Sam James, Megan Conrad, Sankar Sengupta

**Affiliations:** 1 Dept. of Industrial Systems Engineering, School of Engineering and Computer Science, Oakland University, Rochester, MI, USA; 2 Dept. of Mechanical Engineering, College of Engineering and Science, University of Detroit Mercy, Detroit, MI, USA

**Keywords:** Game-based rehabilitation, Grip strength, Home exercise program

## Abstract

Handgrip strength is an essential function of the hand to perform day-to-day tasks. People lose grip strength due to various factors such as aging, diseases, and other medical conditions. According to neuroplastic and physiological principles, grip strength can be improved using goal-oriented tasks or exercises repeatedly and consistently. People often fail to adhere to repeated movements, including grip strength exercises. Studies have shown that game-based rehabilitation has improved exercise compliance and functional outcomes. This article explains the design and development of an affordable smartphone-based telerehabilitation system that includes an innovatively designed grip strength device (eGripper) and a phone application to play games.

Nominal handgrip strength is necessary to grasp a glass of water to bring it from the table to the mouth or open a door. Pathological conditions like stroke, COVID, brain injury, carpal tunnel syndrome, or physiological conditions, such as aging, can affect handgrip strength. When grip strength worsens, performing daily living tasks can become difficult. Resistive exercises can improve grip strength, with the person doing several repetitions. Using multiple repetitions is based on principles of neuroplasticity, which imply that movements should be intensive, repetitive, and task-oriented to regain muscle strength and function. Acute rehabilitation facilities usually focus on gross motor outcomes rather than grip strength or fine motor tasks due to the shorter length of stay imposed by CMS due to restrictions based on Medicare/Medicaid guidelines (Pergolotti et al., 2018). These acute rehabilitation facilities' goals are to get a patient up, to move, to walk, and be discharged to the next level of care. Due to the shorter stay at these acute rehabilitation facilities, there will not be enough days or time to focus on grip strength recovery alone. Health insurance systems, like Medicare and Blue Cross, typically do not reimburse for doing simple exercises like grip strength alone at these high-cost facilities; rather, they will pay for home care or outpatient services (Pergolotti et al., 2018). Therapists typically discharge patients to their homes with instructions to work on repetitive home exercise programs. However, people often fail to comply with home exercise programs (Chhabra et al., 2018).

To improve exercise compliance, a smartphone-based telerehabilitation system, hereafter called the “system,” should mimic clinic settings, including therapists delivering exercises, monitoring progress, and grading exercises based on progress. Evidence shows that in-home telerehabilitation is an effective alternative to in-person rehabilitation (Jack et al., 2010), and a 2015 study reports that nearly two-thirds of Americans use smartphones (Smith, 2020). With the advent of the Internet of Things (IoT, described as a network of physical objects—“things”—embedded with sensors, software, and other technologies for connecting to and exchanging data with other devices and systems via the internet) and communication technologies, delivering home exercise programs via telerehabilitation through a smartphone is feasible (Clark, 2016). Patients should be able to use this system away from the clinical area (e.g., home, assisted living, independent living facilities, coffee shops, libraries) and virtually anywhere. A telerehabilitation system with an innovative device and a customized game was designed to deliver home exercise programs to monitor grip strength and record progress.

## Proposed Telerehabilitation System

**Figure 1:**
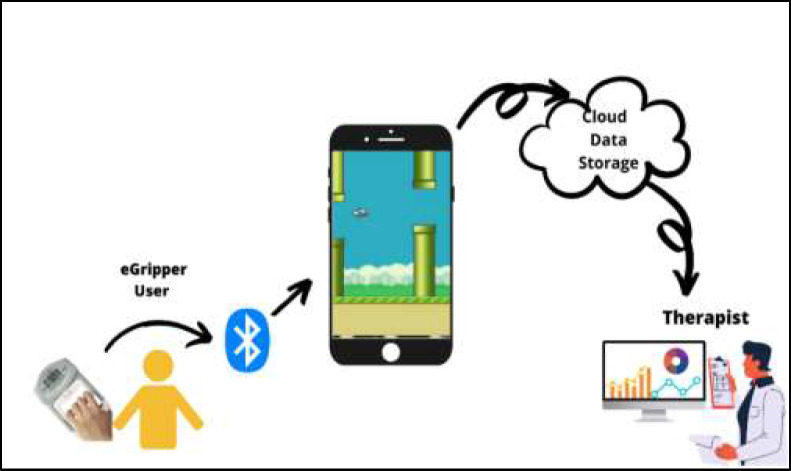
Proposed Telerehabilitation System

Video game-based rehabilitation is fun and motivates patients to engage in home exercise programs (Lange et al., 2009). A system was developed to engage patients through video games using a specially designed sensor-based grip strength exerciser as the game controller (See Figure 1). The system is intended to improve exercise compliance issues by engaging patients in playing games rather than completing relentless repetitive movements. This system challenges patients with poor handgrip strength to play video games using an innovative grip-strength device (eGripper). The game was designed to be customizable for each patient according to their level of weakness, make it playable for all grip strength ranges, and provide incentives for completing their home exercise program.

An administrator dashboard was designed to allow therapists to monitor and track patient progress and modify game parameters to alter the exercise regimen. The dashboard displays patient improvement in clinical parameters, including muscle strength, endurance, usage time, and compliance with the prescribed home exercise program.

## Methods

During the early stages of the design process, formative evaluations were completed, with two participants using the device and answering open-ended questions regarding the device's comfort and gaming operations. Based on their responses, changes were made to include time limits for the game, display points, and grip strength and provide feedback on the grip strength using visual indicators when participants reached target levels. They also suggested that the device handle was very hard to squeeze. Their feedback was implemented during the iterative game design stages. While physical device changes were not feasible at this stage, a soft foam handle was added to increase user comfort.

The research goal was to design and develop an innovative telerehabilitation system incorporating home exercise programs to improve grip strength. The research goal was achieved in three phases: (1) prototype an off-the-shelf hand dynamometer to send grip strength data; (2) design and develop a customized gaming environment and (3) design and develop a dashboard that allows the therapist to monitor grip strength and associated variables, as well as grade exercises/gaming variables.

### Prototype Hardware Design

**Figure 2:**
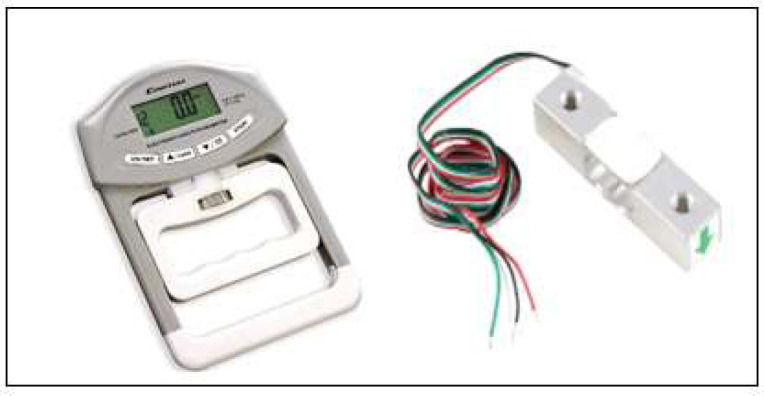
A Commercially Available Hand Dynamometer and Load Cell Inside the Dynamometer

**Figure 3:**
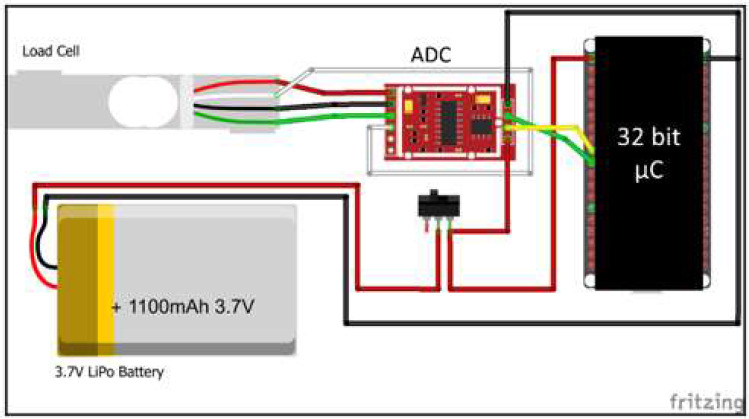
Pre-prototype Assembly

The system's feasibility was determined with a pre-prototype assembly built using breakout modules. A commercially available hand dynamometer (Figure 2), a 32–bit microcontroller prototyping module, a 24–bit ADC, and a lithium battery (Figure 3) were used to construct the pre-prototype module. The electronic hand dynamometer was disassembled, and the existing circuit board was removed, leaving only the load cell, which had a maximum capacity of 220 lbs (Figure 2), to measure the grip strength. The load cell had four wires; each wire was connected to the four nodes of the Wheatstone bridge. An instrumentation amplifier and an analog-to-digital converter (ADC) were used to convert the output from the load cell. The ADC received the input from the load cell, and the ADC's digital output and clock pins were connected to the microcontroller. The microcontroller sent the amplified signal to an android phone through its built-in Bluetooth module. The android phone received the signal from its serial port, with a *Serial Bluetooth Terminal* app by Kai Morich used to read the load cell data. After testing for feasibility, a custom printed circuit board (PCB) was designed as the main prototype module. The PCB was designed in EasyEDA, an online PCB design tool.

**Figure 4:**
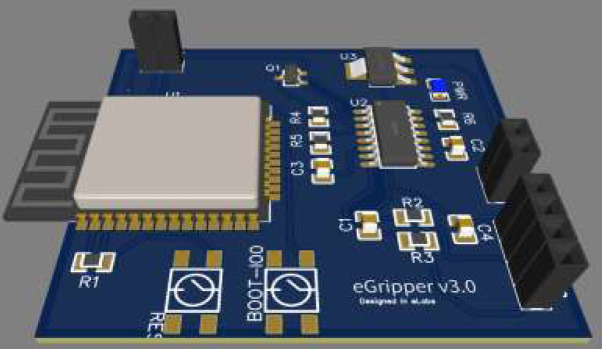
Printed Circuit Board (PCB) Prototype

### Software Design

The software design consists of firmware, a smartphone app, a game, and a dashboard.

#### Firmware Development

The firmware for the microcontroller was programmed in embedded C language and had two purposes. The first purpose was to receive and process the load cell's raw data from the hand dynamometer. The second purpose was to send the processed data to the android phone over Bluetooth communication protocol.

#### App Development

A smartphone app was developed using Apache Cordova (an open-source mobile development framework and web technologies - HTML, CSS, and JavaScript) for feasibility testing deployed for android devices only. The app's primary function was to receive the Bluetooth serial port data from the eGripper and transmit it to the game for character manipulation. The app pushes data into the Google firebase cloud server for storage when the patient plays the game. The administrator's dashboard retrieves the data to display the clinical parameters.

#### Game Development

**Figure 5:**
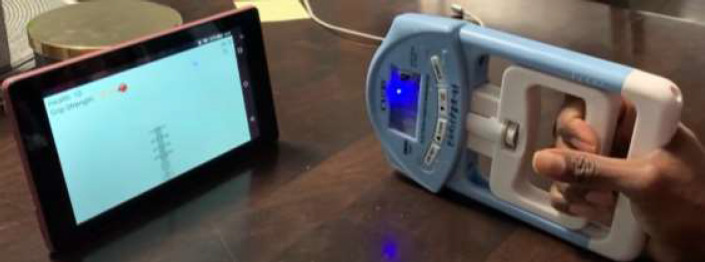
eGripper and grippyBird Game

The game was developed using P5JS, a visual JavaScript library to sketch 2D art. A simple flappy bird-style game (grippyBird) had one level of control (i.e., moving the bird up). When the patient squeezes the eGripper, the load cell data is translated to the grippyBird character to jump up, and gravity will bring the bird down. The game's goal is to make the patient squeeze the eGripper repeatedly to avoid obstacles (e.g., electric posts) to score points. Obstacles come in varying sizes and speeds to keep the patients engaged in the game. During the gameplay, the player's score will be reduced if an obstacle is hit. A customizable time limit can be set to reduce muscle fatigue in patients (Figure 5).

#### Administrator Dashboard Development

The administrator dashboard was programmed with HTML, CSS, and JavaScript web technologies. This dashboard was intended to retrieve the eGripper data and display it as a graph for the frequency of squeezes. In addition, the dashboard also can show the maximum grip strength attained during the session and the number of repetitions performed.

### Calibration

The eGripper's newly designed circuit needed to be calibrated and zeroed before testing. Initially, the raw output from the eGripper was measured without any weights; after that, a few known weights were loaded on the platform, and the raw output with known weights was measured. The initial output without known weights was subtracted from the output with known weights. Since the device's resolution was in three orders of magnitude, minor weight changes were amplified, so the raw output was divided by 1000 to get the correct order of magnitude for the output in pounds. After this correction factor, a few known weights of 0.5, 2.5, 5, 7.5, and 10 pounds were loaded, and their outputs were recorded in Microsoft® Excel. The linear regression was computed with the known weights vs. their output data, and the outputs were in the multiples of five from the regression equation. The final step in processing the raw data was to divide the output by five to yield the corrected values. These calibration and conversion factors were hardcoded in the firmware in the microcontroller for accurate output values.

### Validity and Reliability

#### Concurrent Validity

**Figure 6:**
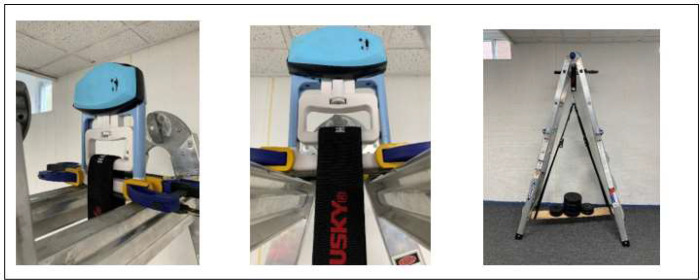
Experimental Setup for Calibrating the eGripper

Validity measures the device's ability to measure what it is intended to measure (Vogt & Johnson, 2016). Concurrent validity is an experiment to study the device's output for validation against the criterion measure. The eGripper was set up for the validation experiment (Figure 6; Fess, 1987). Due to the unavailability of a split-top workbench, a foldable ladder was used to stabilize the eGripper and suspend weights. The eGripper's stationary handle was stabilized on top of the ladder between the top two steps, and a flat piece of a metal bar was placed across the steps. The eGripper was steadied using two bar clamps on each side of the eGripper. All measures were taken to ensure the eGripper's stationary handle was stable, and the movable handle was free to move without obstructions. An easy-hang Velcro strap with a hook was suspended from the moveable handle, where a wooden platform was hung to load weights. The eGripper was zeroed prior to placing the weights on the platform. The eGripper sensed the loaded weights, sending the output to the Android app via Bluetooth. The weights were gradually increased from 0.5 to 130 pounds, with values recorded from the Android app.

#### Inter-Instrumental Reliability

An inter-instrumental reliability experiment was conducted to compare the eGripper device to the Jamar dynamometer, the gold standard in hand grip strength testing. This experiment used healthy subjects to test both devices during the same session. After receiving Oakland University's Institutional Review Board (IRB) approval, 34 healthy participants agreed to participate in the experiment. All subjects were instructed about the experiment and gave informed consent. Each participant completed three trials of their maximum grip strength from both hands, with the grip strength measured from the two devices after each trial. The initial hand used in the test and the device tested were randomized to reduce the fatigue bias. They were asked to squeeze as hard as possible to elicit maximal grip strength and were given tryouts for both devices and sufficient time to recover before starting the experiment. Providing tryouts familiarized subjects with the expectations of the experiment and reduced their anxiety. During the experiment, the participants were given 30 seconds between each squeeze and 2 minutes when changing to the second device to avoid fatigue effects.

### Statistical Analysis

A power analysis was conducted in Minitab® (Minitab Inc. State College, PA) to determine the sample size; using the following settings - the ‘paired t-test,’ difference between means is 2.5 lbs., expected standard deviation set to 5 lbs., power of.80, a two-tailed alpha level of .05, and alternate hypothesis is not equal to H_0_. The power analysis results indicated that 34 subjects were needed to reject the null hypothesis. For the concurrent validity experiment, paired t-test was performed, with a 2.5 lbs. difference between pairs and a criterion 0.05 alpha level. A correlation coefficient (r) was calculated for inter-instrumental reliability.

## Results

**Figure 7:**
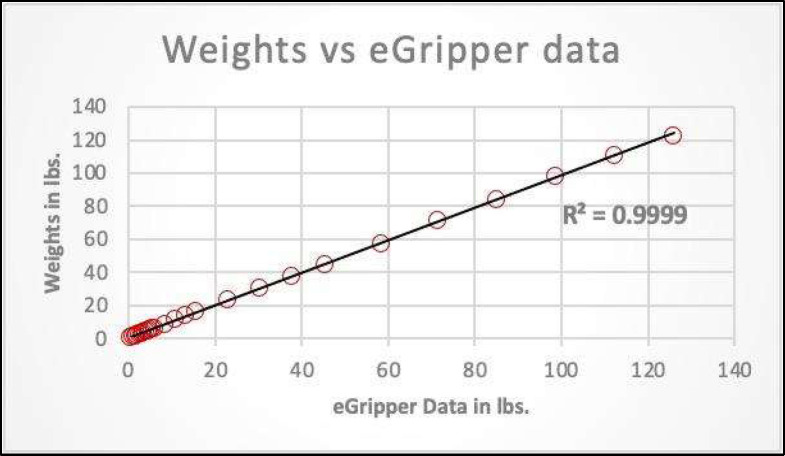
Calibration Data

**Figure 8:**
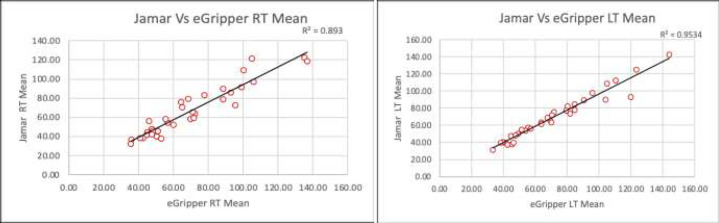
Linear Regression between eGripper and Jamar Dynamometer - Right and Left

**Figure 9:**
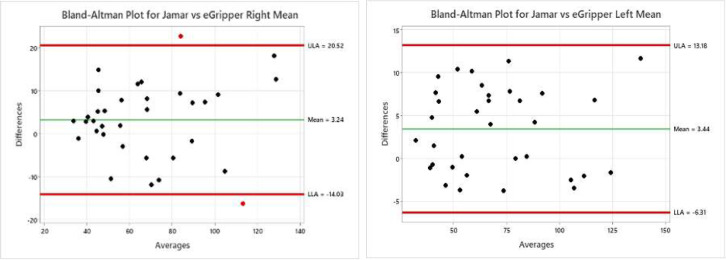
Bland-Altman Plot to Show Agreement between the Two Devices

The known weights versus eGripper data were plotted (Figure 7), and the linear regression line and the r value is 0.9994 was calculated. A paired t-test was performed on the grip strength data between the Jamar and eGripper devices. The testing of paired t-test assumptions for paired differences in normality was performed using Shapiro-Wilk's normality test with no significant difference found from the normal distribution. The p-value for paired t-test for mean grip strengths provided insufficient evidence of a difference between the Jamar dynamometer and eGripper device. The regression analysis provided evidence of a statistically significant positive correlation between eGripper and Jamar dynamometer results (Figure 8). A Bland-Altman plot demonstrated agreement between the two devices (See Figure 9).

**Table 1:** Results of Paired t-Tests between eGripper and Jamar Dynamometers

	Jamar Right	eGripper Right	Jamar Left	eGripper Left
Mean	69.61	66.36	71.33	69.32
SD	26.83	26.12	27.68	26.84
Pearson Correlation	0.94		0.98	
df	33.00		33.00	
t Stat	0.49		−0.47	
P (T<=t) two-tail	0.63		0.64	

## Discussion

The research resulted in the design and development of a prototype of the eGripper, grippyBird game, and administrator dashboard. While other gripper games are on the market, the eGripper provides a unique method for helping patients comply with home exercise programs (HEPs). Espinoza et al. (2016) mentioned remote monitoring of grip strength, but those devices or systems lacked an important feature of telerehabilitation, specifically the feedback or remote adjustments of game parameters. In the present research, that feedback loop was added to keep a patient engaged in the game by adjusting game parameters. The eGripper was able to bridge the gap for remote monitoring and remote gradation of game-based HEPs. The eGripper could be used as a remote grip strength monitoring tool based on validity and reliability findings. Grip strength is essential in carrying out daily living tasks (Bae et al., 2015). To improve hand grip functions and neuroplasticity, patients should do repetitive movements. The practice of using multiple repetitions is based on principles of neuroplasticity, which imply that movements should be intensive, repetitive, and task-oriented to regain muscle strength and function (Demarin et al., 2014). Doing exercises with no objective leads to decreased motivation in completing exercise routines (Chen et al., 1999). The eGripper was found to have good inter-instrumental reliability and was comparable to the gold standard (Jamar dynamometer). The eGripper was calibrated initially to ensure that the grip strength was being measured using the same norms as the Jamar dynamometer that was used for comparison. Frequent calibration was not required because the testing was accomplished in a single session. The concurrent validity was tested with known weights before and after the experiment and with the Jamar dynamometer. The results were indicative of good concurrent validity.

## Limitations

A limitation of the study is the participants who were used to test the eGripper for reliability. The participants were healthy individuals who had no known hand issues that could have limited their grip strength. They were used to determine stability over time. Different results may have been obtained if participants with poor grip strength issues had been included. Additional research will be done to compare healthy individuals and those with grip strength issues to determine reliability.

## Implications

The use of an eGripper could have a profound effect on improving grip strength through repetitive exercise made more appealing with the use of an electronic game. Because repetitive hand grip exercises are boring, most patients fail to complete their HEPs. The eGripper and the flappy bird electronic game provide a more engaging experience for their exercise. Future development can create additional games to improve adherence to HEP. The therapists will have a dashboard that will allow them to monitor the patients' compliance and make changes as the patients' grip strength improves. Additional sensors can be added to the eGripper to increase movements in different planes.

## Conclusion

The outcome of this research was an affordable smartphone-based telerehabilitation system that includes an eGripper device and a smartphone application. The proposed telerehabilitation system was designed to reduce home exercise non-compliance and improve grip strength using this system remotely and supervised by a therapist. This article explained the calibration process with known weights and experiments to evaluate the concurrent validity and inter-instrumental reliability of the eGripper compared with the gold standard Jamar dynamometer. Further development and testing of the eGripper and additional apps will continue to explore the feasibility of telerehabilitation for improving compliance with home exercise programs.
